# Predicting soil thermal properties in freeze-thaw cycles using *EFAttNet*: A comparative analysis

**DOI:** 10.1371/journal.pone.0305529

**Published:** 2024-07-12

**Authors:** Pengcheng Wang, Muge Elif Firat, Yi Lin, Tengfei Wang

**Affiliations:** 1 Railway Engineering Research Institute, China Academy of Railway Sciences Co. Ltd., Beijing, China; 2 Technology Faculty, Civil Engineering Department, Firat University, Elazig, Turkey; 3 College of Computer Science, Sichuan University, Chengdu, China; 4 MOE Key Laboratory of High-Speed Railway Engineering, Chengdu, China; 5 School of Civil Engineering, Southwest Jiaotong University, Chengdu, China; Beijing University of Technology, CHINA

## Abstract

This study investigates the thermal conductivity (*λ*) and volumetric heat capacity (*C*) of sandy soil samples under a variety of conditions, including freeze-thaw cycles at temperatures both above and below zero and differing moisture levels. To estimate these thermal properties, a novel predictive model, *EFAttNet*, was developed, which utilizes custom-designed embedding and attention-based fusion networks. When compared to traditional *de Vries* empirical models and other baseline algorithms, *EFAttNet* demonstrated superior accuracy. Preliminary measurements showed that *λ* values increased linearly with moisture content but decreased with temperature, whereas *C* values exhibited a rising trend with both moisture content and freezing temperature. Following freeze-thaw cycles, both *λ* and *C* were positively influenced by moisture content and freezing temperature. The *EFAttNet*-based model proved highly accurate in predicting thermal properties, particularly effective at capturing nonlinear relationships among the influencing factors. Among these factors, the degree of saturation had the most significant impact, followed by the number of freeze-thaw cycles, subzero temperatures, porosity, and moisture content. Notably, dry density exerted minimal influence on thermal properties, likely due to the overriding effects of other factors or specific soil characteristics, such as particle size distribution or mineralogical composition. These findings have significant implications for construction and engineering projects, especially in terms of sustainability and energy efficiency. The demonstrated accuracy of the *EFAttNet*-based model in estimating thermal properties under various conditions holds promise for practical applications. Although focused on specific soil types and conditions, the insights gained can guide further research and development in managing soil thermal properties across diverse environments, thereby enhancing our understanding and application in this field.

## 1. Introduction

The thermal properties of granular soils play a crucial role in construction and engineering applications, particularly in cold climate regions. These soils typically have a high air void ratio and low water saturation, resulting in their excellent insulating capabilities. The high thermal resistance, high heat capacity, and low thermal conductivity of granular soils are particularly beneficial in reducing the energy required to maintain the internal temperature of buildings. Accurately measuring and considering the heat characteristics of granular soils is essential in reducing the energy consumption of buildings and making them more sustainable. Additionally, the heat characteristics of granular soils are significant factors in other engineering applications, including underground heating and cooling systems. Therefore, studying the thermal properties of granular soils can contribute significantly to improving the efficiency and sustainability of construction and engineering practices in cold climate regions.

In the civil community, there has been a growing interest in understanding infrastructural system performance in cold regions and the frost damages, such as frost heave and thawing subsidence, that occur due to repeated freezing/thawing processes. In recent years, various techniques have been developed and employed to comprehend and address these frost-related issues. These problems are primarily related to thermal properties, which are fundamental parameters for simulating heat transfer in earth structures [1]. A soil’s ability to transfer heat is determined by thermal properties such as volumetric heat capacity, thermal conductivity, and thermal resistivity [2]. Hence, understanding and accurately measuring these thermal properties are essential in effectively addressing frost-related issues in cold regions.

The design of engineering structures, energy foundations [3–9] (e.g. piles used as a ground-source heat exchanger), deep underground radioactive waste disposal [10–12], and underground power cables [13, 14], is highly dependent on soil thermal properties. The thermal properties of soils are affected by both intrinsic and extrinsic soil parameters. The mineralogical compounds present in the soil constitute intrinsic parameters [15, 16], while external parameters such as temperature and freeze-thaw processes have been recognized as crucial factors that affect soil heat transfer [17–23]. Hence, a thorough understanding and precise measurement of soil thermal properties are imperative for designing effective and sustainable engineering structures.

The soil moisture content plays a significant role in determining the heat transfer through soil; however, managing it in soil systems poses a significant challenge [21, 24]. In areas with seasonal freezing, soil water content is highly influenced by temperature, and it frequently undergoes phase transitions between solid and liquid states. This results in variations in the soil’s thermal properties since ice has a higher thermal conductivity than water [25]. Furthermore, soil particle shape and granulometry variations make the freeze-thaw process a critical factor in soil’s thermal behavior. The topsoil in such regions undergoes many freeze-thaw cycles each year, leading to severe damage to infrastructure and superstructure above the soil foundation. This damage includes issues such as structural instability, surface layer heave, and fractures in highway asphalt layers [26, 27].

Various methods are used to determine the thermal properties of soils, including in-situ and laboratory techniques, numerical analysis, and empirical models. Empirical models, in particular, have become prevalent and effective in this domain. They estimate thermal properties using various soil parameters, such as soil water content, porosity, mineralogical composition, and moisture status, along with temperature, humidity, and soil type and properties. These models are typically validated with experimental data, often yielding favorable outcomes. Their value is especially notable in geotechnical and railway engineering, where they assist in estimating thermal conductivity and heat capacity. This information is crucial for designing and constructing safe and reliable infrastructure [28–33].

In addition to traditional methods, neural networks and computer-based models have become increasingly prominent in estimating soil thermal properties. One study introduced a model utilizing artificial neural networks to estimate soil thermal conductivity by considering variables such as moisture content, dry density, soil mineralogy, and structure [31, 32]. Pan et al. [34] evaluated the performance of various soil thermal conductivity (STC) schemes in predicting soil temperature and permafrost extent, comparing nine typical STC schemes with laboratory measurements from undisturbed soil samples. These schemes were integrated into the Community Land Model (CLM5.0) to assess their effectiveness in simulating soil temperature and permafrost extent in the Tibetan Plateau region, with the default scheme performing moderately well, ranking third among the evaluated schemes. This research underscores the significant influence of different STC schemes on permafrost dynamics simulation and the importance of considering uncertainties in land surface models, including atmospheric forcing, soil moisture, and soil texture, to make accurate permafrost predictions. Ji et al. [35] proposed a unified model for STC based on the Ghanbarian and Daigle [36] framework, originally designed for unfrozen soils, that integrates three parameters: critical volume fraction, scaling exponent, and a compensating factor, which account for both dry and saturated soil components. Pedotransfer functions (PTFs) were developed using a dataset of unfrozen and frozen soil samples, enabling the unified model to successfully capture variations in STC under different moisture conditions and freezing states, outperforming existing empirical and theoretical models. This research enhances the understanding of STC in cold environments and offers potential integration into land surface models. Wang et al. [37] utilized machine learning (ML) algorithms to integrate soil thermal conductivity, matric potential, and water content, employing a dataset of 2328 measurements from 43 soils to train, validate, and test five ML algorithms: RF, GBDT, SVM, LR, and BPNN. The results showed that ML algorithms outperformed empirically based methods in optimizing the thermal and hydraulic relationship, with GBDT performing best for small soil hydrothermal datasets, while BPNN showed limitations due to data size constraints. Additionally, Cheng et al. [38] introduced a novel method for measuring soil thermal conductivity using Optical Frequency Domain Reflectometry (OFDR), which offers advantages such as distributed measurement, high accuracy, and fast speed compared to traditional methods. The study investigated the impact of various factors on soil thermal conductivity using sand as a case study, revealing an "S"-shaped growth trend in soil thermal conductivity with increasing water content in non-seepage conditions, whereas seepage resulted in higher thermal conductivity values with a positive linear correlation to seepage velocity. The OFDR method showed high accuracy relative to other techniques. Furthermore, Dong et al. [39] developed an artificial neural network model to predict soil thermal conductivity in the field, considering soil properties and groundwater characteristics, and introduced a new system to predict in-situ thermal conductivity. Cui et al. [40] developed and compared various neural network models for soil thermal conductivity, including radial basis functions, binary fitting, and ternary fitting predictive models, in both frozen and unfrozen states.

This research explored how temperature, moisture content, porosity, dry density, saturation, and freeze-thaw cycles impact soil thermal properties (*λ* and *C*). To predict these properties, *de Vries* empirical models were used, and their performance was compared. Additionally, a new deep neural network (*EFAttNet*) was developed, using a thermal properties database to predict soil thermal properties. The study provides critical insights into how granular soil transfers heat when exposed to freezing and thawing cycles, and it offers theoretical guidance for calculating the heat of frozen soil. Accurate measurement and calculation of thermal properties are essential to reduce energy consumption and build more sustainable structures in the future. This study will be beneficial to those working in construction, engineering, sustainability, and experts in soil mechanics and geotechnics.

## 2. Materials and methods

This section outlines the comprehensive methodologies used to investigate the thermal properties of soil samples. Beginning with the examination of the soil’s basic properties, the study employs a range of standardized tests and procedures. It then progresses to assess the impact of freeze-thaw cycles on these properties, utilizing specialized analytical equipment. The section is structured to provide a systematic approach for obtaining accurate and reliable data on soil behavior under varying thermal conditions.

### 2.1 Study area and specimens

This study aims to investigate soil samples gathered from Elazig Province in eastern Turkey. Elazig Province, with a cold continental climate, is prone to frost-related issues due to harsh winter weather and considerable precipitation, as stated in Table 1.

**Table 1:** Climatic conditions of the study region.

Index	Values	Units
Average Precipitation Rate	130.4	mm
Freezing Period	November—March	-
Maximum Snow Depth	3.0	m
Count of Snow-Covered Days	120	days
Minimum Temperature in Coldest Month	−8.57	°C
Surface Freezing Index	211.47	°C-day
Frost Depth	60–140	cm

**Note**: Surface Freezing Index: The cumulative sum of negative daily mean air temperatures (below 0°C) over a cold season.

**Fig 1:**
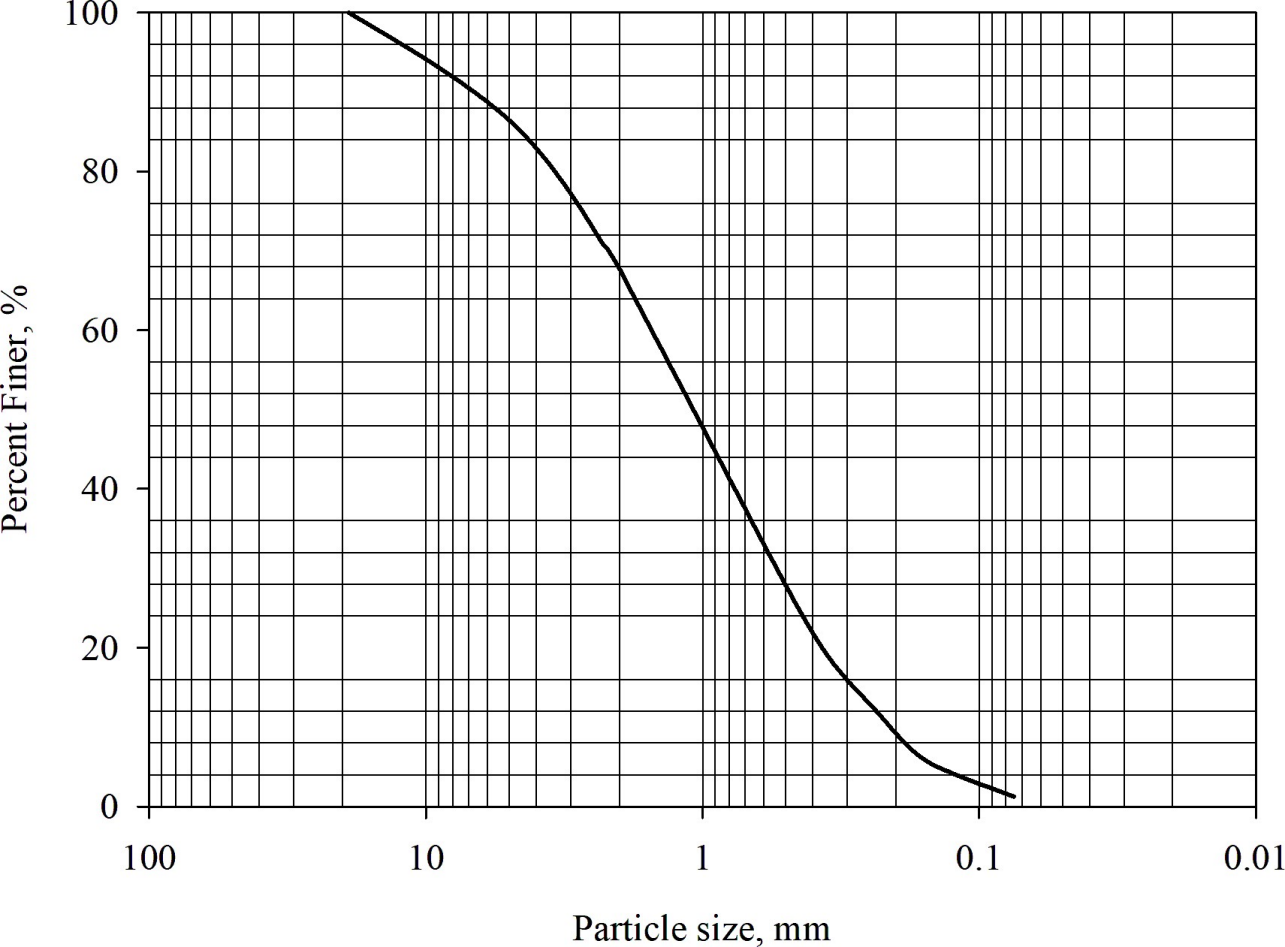
Soil particle size distribution.

The evaluation of the soil’s thermal properties was conducted by determining its index properties. The findings indicated that the maximum dry density was 1.65 g/cm^3^, and the optimum moisture content was 24.62%, according to the ASTM D698-00 standard [41]. Additionally, the particle size test [42] revealed that the mass of particles smaller than 0.075 mm accounted for only 1.266% of the total mass. Fig 1 displays that, according to the Unified Soil Classification System [43], the soil was classified as well-graded sand (SW) with a coefficient of uniformity of 7.81 and curvature coefficient of 0.937.

To assess the thermal properties (*λ* and *C*) of the soil specimens, a total of 119 cylindrical specimens were prepared with a diameter of 10.2 cm and a height of 11.6 cm at six different volumetric moisture content values (*θ*) ranging from 0.190 to 0.320 m^3^/m^3^. Additionally, to investigate the effect of moisture content on thermal properties, specimens were also created at an optimum moisture content ratio (OWC) of 0.246 m^3^/m^3^, as well as at lower and higher moisture content ratios (0.190 m^3^/m^3^, 0.212 m^3^/m^3^, 0.230 m^3^/m^3^, 0.260 m^3^/m^3^, and 0.320 m^3^/m^3^). The soil specimens were dried uniformly in an oven at approximately 110°C for 12 hours prior to preparation to ensure homogeneity and minimize evaporation. The specimens were then placed in a proctor mold and compacted into three layers. Finally, to prevent moisture loss, the specimens were wrapped with plastic and stored in a humidity-controlled cabinet.

### 2.2 Freeze-thaw procedures

**Fig 2:**
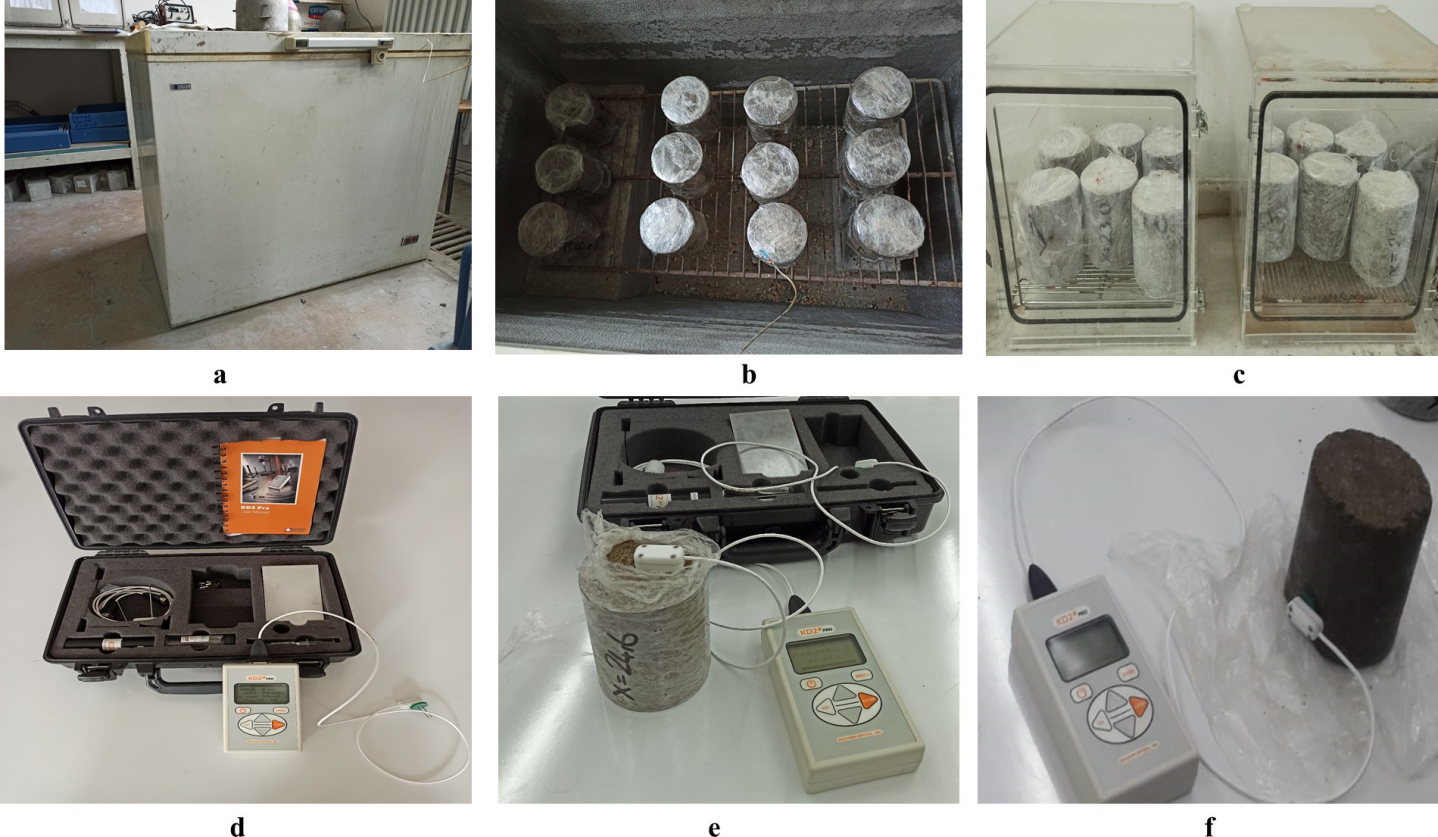
Testing Overview: (a) Environmental Chamber; (b) Freezing Process; (c) Thawing Process; (d) Thermal Testing Apparatus; (e) Thermal Testing Procedure 1; (f) Thermal Testing Procedure 2.

The thermal characteristics of soil samples were determined by subjecting them to a closed-system freezer for 0, 2, 5, 9, and 12 cycles of freezing and thawing. Prior to thermal assessments, the specimens underwent freeze-thaw testing following the ASTM D5918 standard [44]. The procedure involved gradually decreasing the temperature of the samples to 4, 0, −7, −12, and −20°C for 24 h. Freeze-thaw tests were conducted at each sub-zero temperature value to determine the thermal properties of the specimens. To achieve thermal equilibrium between the samples and the ambient environment, the temperature in the freezer was maintained for 12 hours before each freeze-thaw cycle. After each freezing and thawing cycle, the specimens were placed in a humidity-controlled chamber at ambient temperature for 24 h to thaw. Table 2 and Fig 2 provide detailed information on the test cases and the preparation of soil specimens for thermal testing.

**Table 2:** Metrics for thermal tests and exposure temperature control.

ID	Moisture Content (m^3^/m^3^)	Dry Density (g/cm^3^)	Temp. (°C)	Thawing Temp. (°C)	Number of F-T Cycles
S1	0.190	1.558	–20, –12, –7, 0, +4	20	0, 2, 5, 9, 12
S2	0.212	1.616	–20, –12, –7, 0, +4	20	0, 2, 5, 9, 12
S3	0.230	1.642	–20, –12, –7, 0, +4	20	0, 2, 5, 9, 12
S4	0.246	1.649	–20, –12, –7, 0, +4	20	0, 2, 5, 9, 12
S5	0.260	1.642	–20, –12, –7, 0, +4	20	0, 2, 5, 9, 12
S6	0.320	1.612	–20, –12, –7, 0, +4	20	0, 2, 5, 9, 12

### 2.3 Thermal testing methodology

The KD2 Pro Thermal Properties Analyzer (Decagon Devices) [45], depicted in Fig 2D, was utilized to establish the heat characteristics of the soil specimens. The thermal characteristics of the specimens were evaluated with a precision of 0.02 W/(m·K) using the transient approach principle. The SH-1 thermal sensor (0.13 cm diameter, 3 cm long, and 0.6 cm spacing) with a dual needle was utilized for thermal property measurements. This sensor uses a combination of infinite line heat pulse method and transient method to determine thermal properties. The transient method has an advantage of reducing the impact of temperature on moisture migration in specimens [46]. The SH-1 sensor was designed to operate in low-power mode for measurements in a frozen medium, which reduces heat input and minimizes the possibility of phase change in the frozen specimen. Prior to conducting the experiments, the thermal sensor was calibrated. The SH-1 needle was used under default settings, which included a reading taken every two minutes and high-power mode.

Sensors were positioned within the specimens at three different locations: the bottom, middle, and top, to record measurements. Subsequently, the average thermal properties of each specimen were computed. The heat characteristics of the soil specimens were then measured following the addition of water to the soil [47]. For the measurements, thermal sensors were positioned both horizontally and vertically. Before the commencement of the measurements, the soil specimens were stabilized at a constant temperature to maintain the soil surface temperature and avoid evaporation. To determine the thermal properties of frozen soil, the unfrozen properties were initially assessed. Next, the specimens were frozen overnight in a freezing cabinet at freezing temperatures while an implanted needle was used to minimize disturbance to the specimens. This technique was determined to have an error rate of less than 1% [48].

A needle containing a heater and temperature sensor was utilized in the heat pulse method to form a system for conducting the test. The short heating time was designed to minimize thermally induced moisture migration and decrease the duration of the measurement. During the testing, heat generation was limited to minimize water flow and convection. The KD2 Pro was used to achieve high-resolution temperatures of ±0.0001°C, which was necessary for utilizing the minimized heating time and low heating rates.

The thermal testing method involved applying heat to a heated needle for a specific period (*t*_h_) and monitoring the temperature at 6 mm in the monitoring needle during both the heating and cooling phases. The test results were analyzed using nonlinear least-squares equations:

$$T=b_{0}t+b_{1}Ei\left(\frac{b_{2}}{t}\right)$$ (1)

$$T=b_{0}t+b_{1}\left\{Ei(\frac{b_{2}}{t})-Ei[\frac{b_{2}}{t-t_{h}}]\right\}$$ (2)

$$T=\frac{4\pi (T_{1}-T_{0})}{q}$$ (3)

where *b*_2_, *b*_1_, and *b*_0_ are the constants used for fitting, *E*_i_ represents the exponential integral, *T*_0_ is the initial temperature during the measurement, and *q* denotes the amount of heat input. Eq (1) is applicable when the heat is being applied for the first *t*_h_ second, whereas Eq (2) is valid when the heat is turned off [49]. In addition, to obtain the readings, the ambient temperature at time 0 must be subtracted, multiplied by 4π, and divided by the heat per unit time (*q*), as shown in Eq (3).

Several factors can impact experimental uncertainty, including device selection and calibration, experimental setup and design, and data collection techniques. These uncertainties are crucial for the applicability of experimental results. In this study, the Root-sum-square (RSS) approach, a commonly used method for error analysis in engineering applications, was utilized to assess uncertainty [50]. According to the RSS approach, the experimental uncertainties for the thermal conductivity and heat capacity of the soil specimen with a water content of 0.190 m^3^/m^3^ are 0.0181% and 0.0173%, respectively. For the specimen with 0.230 m^3^/m^3^ water content, the uncertainties are 0.0179% and 0.0172%; for the one with 0.246 m^3^/m^3^, they are 0.0176% and 0.0172%; for the one with 0.260 m^3^/m^3^, they are 0.0174% and 0.0172%; and for the specimen with 0.320 m^3^/m^3^ water content, the uncertainties are both 0.0171%. The total error for each measurement consists of quantifiable errors from various sources such as temperature non-uniformity of the device, calibration, data collection, data processing, and signal conditioning. Additionally, non-quantifiable variables like water redistribution in the soil layer due to gravity and thermal gradients, as well as the homogeneity of soil specimens, may also influence the results.

## 3. Predictive models for soil thermal properties

Drawing from the experimental findings, we introduce and evaluate two methodologies for predicting soil thermal properties. These approaches are further scrutinized for their viability in real-world applications. As indicated in Table 2 and corroborated by test outcomes, it should be noted that the key influencing factors, which are already integral to both semi-empirical and theoretical models, have been incorporated into the development of data-driven models to improve the prediction of soil thermal attributes.

### 3.1 *de Vries* model

This section utilized the models proposed by *de Vries* [2] to forecast the values of *λ* and *C* for the examined soil under varying conditions and compared the results with those of the deep neural network-based model described in the following sections. To estimate the heat capacity and specific heat of the soil, *de Vries* [2] and Bristow [23] employed empirical formulas based on laboratory experiments. The constituents of frozen soil consist of four elements: soil particles, ice, water, and voids. Soil grains are generally encased in a thin layer of unfrozen liquid, and their sizes and shapes vary. The voids are typically filled with air, dust, and unfrozen water. The heat diffusion equation given in Eq (4) for a soil specimen at temperature *T*, height *t*, and time *z* is generated by soil heat transfer:

$$c\frac{\partial T}{\partial t}=\frac{\partial }{\partial z}\left(\lambda \frac{\partial T}{\partial z}\right)+\rho_{ice}L_{f}\frac{\partial \theta_{ice}}{\partial t}$$ (4)

where *ρ*_ice_ is the ice density, and *θ*_ice_ is the volumetric ice content. *de Vries* [2] derived an equation for calculating the volumetric heat capacity of frozen soil, which is expressed as follows:

$$c=c_{s}(1-\theta_{sat})+\theta_{liq}c_{liq}+\theta_{ice}c_{ice}$$ (5)

where *c*_s_, *c*_liq_ and *c*_ice_ are the volumetric heat capacity of the soil solids, unfrozen water and ice, respectively.

The determination of *λ* relies on the Maxwell equation, which is the basis of the *de Vries* model [2]. The *de Vries* model makes certain assumptions for predicting thermal conductivity, namely: (i) the soil is a two-phase material, and (ii) the soil is composed of homogeneous solid particles with elliptical shapes. The equation below is employed to compute the thermal conductivity of the soil:

$$\lambda =\frac{f_{0}\lambda_{0}+\sum_{n=1}^{N}k_{n}f_{n}\lambda_{n}}{f_{0}+\sum_{n=1}^{N}k_{n}f_{n}}$$ (6)

where ƒ_0_ and ƒ_n_ the volumetric fractions of the continuous media and the ellipsoidal grains, respectively; *λ*_0_ and *λ*_n_ are the thermal conductivities of the continuous media and ellipsoidal grains, respectively; *N*: the amount of grain type; *k*_n_ is the weighting parameter and calculated as the following equation:

$$k_{n}=\frac{2}{3}{\left[1+(\frac{\lambda_{n}}{\lambda_{0}}-1)g_{a}\right]}^{-1}+\frac{1}{3}{\left[1+(\frac{\lambda_{n}}{\lambda_{0}}-1)(1-2g_{a})\right]}^{-1}$$ (7)

where *g*_α_ is the shape parameter of ellipsoidal grains and computed by:

$$g_{a}=\left\{ \begin{array}{l} 0.333-x_{\frac{a}{n}}(0.333-0.035)for0.09\leq x_{w}\leq n\\ 0.013+0.0944x_{w}for0\leq x_{w}\leq 0.09 \end{array}\right.$$ (8)

The *de Vries* model was extended by Farouki [51] to simulate thermal properties of frozen soils. In this model, it is presumed that the soil grains are of a single type and the water in the soil is a continuous media.

$$\lambda =\frac{\theta_{water}\lambda_{water}+k_{ice}\theta_{ice}\lambda_{ice}+k_{air}f_{air}(\lambda_{air}+\lambda_{vapour})+k_{m}f_{m}\lambda_{m}}{\theta_{water}+k_{ice}\theta_{ice}+k_{air}f_{air}+k_{m}f_{m}}$$ (9)

where ƒ_m_, ƒ_air_ *θ*_ice_, and *θ*_water_, are the volumetric ratios of minerals, air, ice, and liquid water, respectively; *k* is the weighting factors of the components; $\lambda_{vapour}=\lambda_{vapour}^{s}$ for 0.09 m^3^ m^-^^3^ ≤*θ*_water_ ≤ *θ*, and $\lambda_{vapour}=\frac{\theta_{\mathrm{water}}}{0.09}\lambda_{vapour}^{s}$ for volumetric ratios of water; *θ* is the soil porosity.

### 3.2 Deep neural network-based model

#### 3.2.1 General overview

Experimental data obtained from thermal tests were utilized to generate a comprehensive database, identifying volumetric heat capacity and thermal conductivity as two primary outputs. The six input variables or predictors considered were ambient temperature, soil moisture content, porosity, dry density, degree of saturation, and the number of freeze-thaw cycles. The compiled database serves as a crucial resource in enabling predictions of soil thermal properties through data-driven approaches.

To facilitate accurate predictions of soil thermal properties, a cutting-edge deep neural network-based model has been developed. This innovative model, dubbed *EFAttNet*, is specifically designed to capture nonlinear dependencies among the input measurements gathered under various working conditions. *EFAttNet* leverages an independent embedding and attention-based fusion network to effectively synthesize and analyze the data. The model is comprised of several modules that work cohesively to deliver robust predictions, as detailed below.

- *Embedding Module*: Given the distinctive contributions of various attributes, an independent embedding module has been developed. This module is tasked with extracting abstract high-level representations for each input attribute, ultimately providing informative orientations that are vital for the prediction task at hand.
- *Fusion Module*: The hidden features generated by the embedding module are subsequently fed into the fusion model, where they are utilized to generate an integrated fused representation. The primary objective of this process is to uncover the shared correlations that exist among the various raw attributes.
- *Prediction Module*: The prediction module is responsible for generating accurate predictions for the output items, such as thermal conductivity, based on the fused representation. By achieving this critical prediction task, the *EFAttNet*-based model becomes an invaluable tool for supporting decision-making within the construction industry, particularly for projects that operate in cold regions.

In the proposed model, the fully connected (FC) layer serves as the main block, given the discrete options available for the input attributes. The inference rules of the FC layer can be precisely defined mathematically as follows:

$$z_{j}^{l}=\sum_{k=1}^{m}w_{jk}^{l}a_{k}^{l-1}+b_{j}^{l}$$ (10)

$$a_{j}^{l}=f(z_{j}^{l})$$ (11)

where *l* is the layer index and *j* is the index of the neuron. There are *m* neurons in Layer *l*−1. $a_{k}^{l-1}$ is the input *k*^*th*^ neuron in Layer *l*−1. $w_{jk}^{l}$ saves the trainable weight for a specific neuron connecting Layers *l* and *l*−1. $b_{j}^{l}$ is a learnable bias to support the model optimization. $z_{j}^{l}$ denotes the nonactivated outputs of the *j* in Layer *l*. *f*(*) is an activation function to enhance the nonlinear modeling ability. The widely used activation function can be selected from *Sigmoid*, *tanh*, *ReLU*, etc.

**Fig 3:**
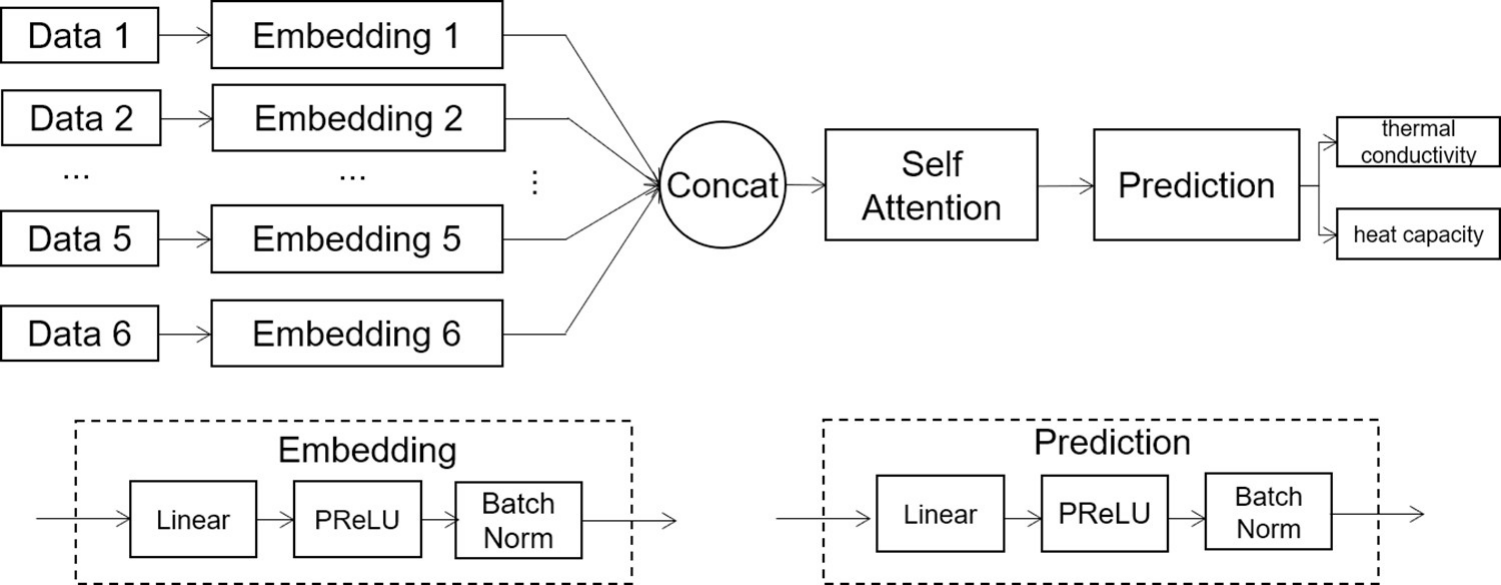
Architecture of the proposed deep neural network model.

In addition, a hierarchical architecture is employed to stack the FC layers, enabling the neural network to iteratively optimize its trainable parameters to achieve a better fit for the data distribution between the inputs and outputs. A comprehensive illustration of the complete model architecture is presented in Fig 3.

#### 3.2.2 Embedding module

The embedding module comprises several independent networks that transform scalar-based inputs into an embedding vector. The objective of this module is to extract input-related information independently to enhance the representation capability of the hidden features. The proposed model includes six embedding modules, each with unique trainable parameters optimized by a data-driven mechanism and sharing the same architecture.

The input format dictates the selection of FC layers as the primary building blocks in the embedding modules for this study. Specifically, one FC layer is stacked for each input, with batch normalization and activation layers (*PReLU*) to enhance training convergence and nonlinear modeling capabilities. Ultimately, the output for each input is a set of 32-dimensional hidden features, which are then fed into the fusion modules that follow.

#### 3.2.3 Fusion module

**Fig 4:**
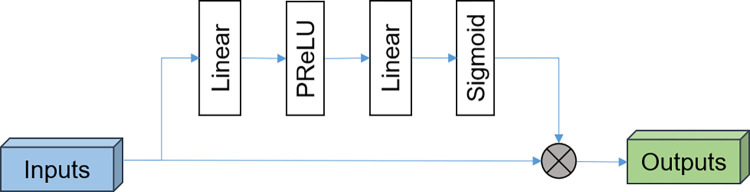
Self-attention mechanism architecture in deep neural networks.

To obtain informative representations based on the embedding vectors, a fusion module is designed, considering the correlations among the input items. The fusion procedure is carried out in the following steps: To formulate a unified embedding vector of 192 dimensions, a concatenation operation is performed as the first step of the fusion procedure. Additionally, it is believed that considering the correlations among the obtained embeddings is essential for this task, and thus, an attention-based fusion block is proposed in this module to capture the hidden correlations among the dimensions of the unified embedding. A self-attention mechanism is utilized to perform feature fusion, where the model architecture is depicted in Fig 4.

As seen from the Fig 4, an attention weight vector is randomly initialized from a uniform distribution to serve as a base weight, i.e. *W*_*l*_∈ℝ^*L*×1^ (*L* is the length of the unified embedding), which is trainable with the training process. In succession, two FC layers (with ReLU activation) are designed to fine-tune the attention weights in a data-driven manner, as shown in Eq (12). A sigmoid activation function is applied to ensure that the weights for all dimensions are summed to 1.0.

$$W_{Att}=g_{Att}(W_{I}),W_{Att}\in R^{L\times 1}$$ (12)

$$W_{Att}=Sigmoid(W_{Att})$$ (13)

where *g*_*Att*_ is the inference rule of the fine-tuning network, and *W*_*Att*_ is the optimized attention weight. The attention mechanism assigns a scalar weight to each dimension of the unified embedding to denote their contributions to the final prediction task.

Finally, by multiplying the input unified embedding and the learned attention weights, the fine-tuned attentive vector (i.e. *F*_*Att*_) is obtained to complete the fusion procedure.


$$F_{Att}=W_{Att}F,F_{Att}\in R^{L\times 1}$$ (14)

#### 3.2.4 Prediction module

Upon completing the intricate process of extracting hidden representations, a prediction module is appended to accurately forecast the final outputs: thermal conductivity and heat capacity. The input of the prediction module is the attentively fine-tuned vector while the output is the output items. The prediction module’s output dimension is 2, where the two dimensions represent the thermal conductivity and heat capacity, respectively. To optimize the prediction module, three fully connected layers were designed, containing 32, 6, and 2 neurons. The model’s convergence is enhanced by including batch normalization layers, and the activation function of the final layer is ReLU, which eliminates any negative prediction values.

By combining and stacking the modules, the proposed model aims to accurately predict the desired outcome. The loss function selected for optimizing the model is the mean square error (MSE), which measures the discrepancies between the predictions and the ground truth. Eq (15) shows the formula, where *N* is the batch size, *y*_i_ represents the ground truth, and ${\hat{y}}_{i}$ represents the prediction for the *i*^*th*^ specimen.


$$MSE=\frac{1}{N}\sum_{i=1}^{N}{\left(y_{i}-{\hat{y}}_{i}\right)}^{2}$$ (15)

Since the proposed model predicts two outputs, the final loss is illustrated by Eq (16), where *MSE*_*tc*_ and *MSE*_*hc*_ denote the MSE loss for the thermal conductivity and volumetric heat capacity, respectively.


$$L=MSE_{tc}+MSE_{hc}$$ (16)

The code generated for this study is available at 10.6084/m9.figshare.25922419 to facilitate reproducibility and reuse.

## 4. Results and discussion

This section presents the outcomes of laboratory experiments, as well as empirical and predictive models, along with their performance evaluations. Specifically, the performance of the novel *EFAttNet*-based model is compared to that of baseline models, as well as the *de Vries* model, to showcase its efficacy.

### 4.1 Volumetric unfrozen water content

Frozen soil is a complex structure consisting of soil particles, voids, ice, and water. Soil particles vary in shape and size, typically covered by unfrozen water, while voids contain dust, air, and unfrozen water. The thermal conductivity (*λ*) of frozen soil is significantly affected by these components, especially unfrozen water and ice. Unfrozen water plays a key role in determining λ as the total quantity of soil particles and water remains constant. Initially, during freezing, liquid water is abundant, but as temperature decreases, unfrozen water diminishes, leading to increased thermal conductivity. Understanding these changes in unfrozen water and ice content at different subfreezing temperatures is crucial for comprehending soil strength and thermal properties in seasonally frozen regions [52, 53].

Various empirical techniques exist for determining the unfrozen moisture content in soil, typically incorporating parameters like soil temperature, soil water curves, different water types, and soil particle surface area [5, 54–57]. One such equation proposed by Anderson and Tice [54], and Anderson and Morgenstern [55], denoted as Eq (17), establishes the relationship between subzero temperature and unfrozen water content.


$$w_{u}=\frac{\rho_{s}(1-w_{s})}{100\rho_{w}}\alpha (-T{)}^{\beta }$$ (17)

**Fig 5:**
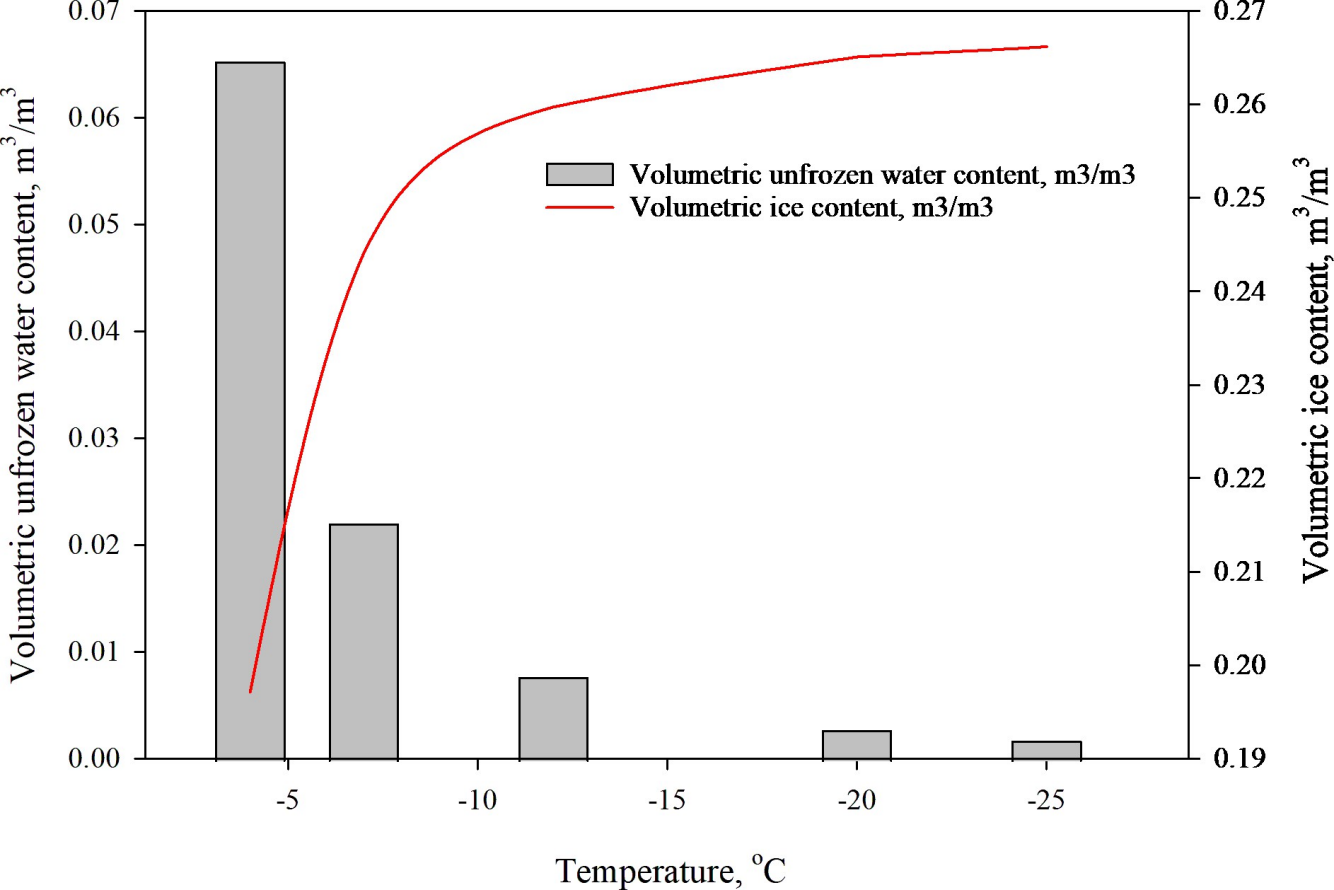
Relationship between unfrozen water content and ice content vs. sub-zero temperature.

Eq 17 consists of the volumetric moisture content (*w*_u_), soil particle density (*ρ*_s_), and water density (*ρ*_w_), along with two regression parameters, *α* and *β*. These regression parameters are determined through logarithmic regression analysis of the data and have been prescribed as 0.948 and 1.930, respectively. Eq (18) is used to calculate the variations in the amount of ice in a given volume (measured as *θ*_i_ in m^3^/m^3^) at temperatures below zero, and these calculations are illustrated graphically in Fig 5.

$$\theta_{i}=\frac{\rho_{w}}{\rho_{i}}(w-w_{u})$$ (18)

where *ρ*_w_ and *ρ*_i_ are the densities of water and ice, respectively, and *w* is the optimum moisture content of the soil (m^3^/m^3^).

Fig 5 shows the relationship between soil temperature and the amount of unfrozen water content in a soil volume. As the temperature approaches the freezing point, the soil contains a substantial quantity of liquid water. Even a slight temperature variation can cause a notable change in the quantity of unfrozen water content, which can significantly affect the *λ* and *C* of soil. For soils with fine particles (defined as those having less than 1.27% mass < 0.075 mm), the frozen soil’s heat properties are particularly sensitive to changes in unfrozen moisture content. The unfrozen water quantity in the soil diminishes exponentially as the temperature decreases. Soil heat transfer occurs in four phases at subzero temperatures, which include solid mineral particles, ice, water, and gas. As the temperature drops, water in the soil gradually freezes into ice crystals.

### 4.2 Impact of moisture content, freeze-thaw cycles, and temperature on soil thermal properties

**Fig 6:**
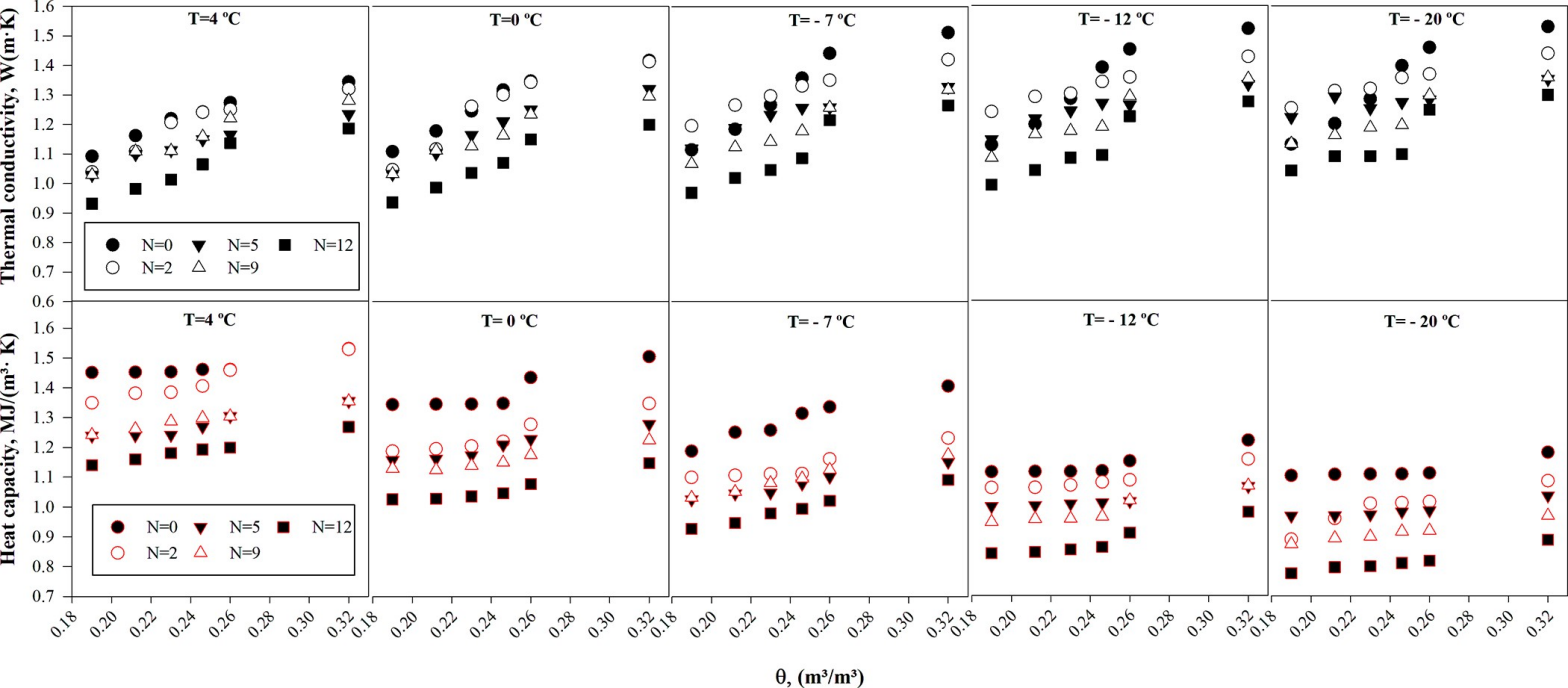
Variability in soil thermal properties across different moisture levels and freeze-thaw cycles for various freezing temperatures.

The thermal properties of soil are determined by various factors, including moisture content, temperature, and the freeze-thaw cycle, as depicted in Fig 6. Previous studies [22, 58–60] have reported that the soil’s thermal conductivity increases with increasing dry density (*ρ*_d_) and moisture content (*θ*). The dry density reaches its maximum value at the soil’s optimum moisture content (OWC), while the dry density decreases with an increase in moisture content. The *λ* values were found to range from 1.092 to 1.397 W/(m·K) as the dry density increased and the temperature dropped from 4 to −20°C.

As the temperature decreased from 4 to −20°C, *λ* values decreased as *ρ*_d_ decreased, as shown in previous research [22, 52–54]. Specifically, *λ* values ranged from 1.274 to 1.529 W/(m·K) during this temperature range. Additionally, exponential growth was observed before freeze-thaw cycles for all soil specimens as the temperature fell below the freezing point. Soil specimens experienced an increase in *λ* values as the temperature decreased, owing to the higher thermal conductivity of ice, which is four times greater than that of water. Another observation was that faster heat transfers occurred in the soil texture when soil porosity decreased due to the formation of strong bonds between soil grains. Lastly, for all volumetric moisture content values, the *λ* values stabilized at extremely low temperatures, likely due to the formation of ice.

As the temperature decreases, the ice mass in the soil structure increases, enhancing the adhesive force and promoting more effective heat transfer through emerging ice cementation. The soil porosity serves as a crucial factor in the heat transfer of soil structure, particularly in the contact area of particles. In terms of volumetric heat capacity, higher dry density resulted in *C* values ranging from 1.103 to 1.461 MJ/(m^3^·K) as the temperature declined from 4°C to −20°C, while decreasing *ρ*_d_ led to *C* values ranging from 1.112 to 1.531 MJ/(m^3^·K). Moreover, volumetric heat capacity increased with the rise in volumetric moisture content and decreased as the temperature decreased (as presented in Fig 6). An increase in soil moisture content (from 0.190 m^3^/m^3^ to 0.280 m^3^/m^3^) results in the filling of the soil’s pores with water, thereby reducing the amount of air in the pore space. This is crucial, as the volumetric heat capacity of water (0.597 W/(m·K)) is substantially greater than that of air (0.026 W/(m·K)), which influences the overall thermal properties of the soil. Finally, the specimens displayed a declining trend in volumetric heat capacity as the temperature decreased to freezing temperatures.

The thermal behavior of granular soils, such as gravel and sandy soils, is significantly impacted by the seasonal freezing and thawing processes. With the onset of freezing, ice lenses start forming and growing in the soil structure at lower temperatures, leading to alterations in the soil density. The presence of these ice lenses within the soil’s structure greatly influences the soil’s heat transfer capabilities throughout the freezing phase. In sandy soils, impervious formations were identified after the freezing process, and during the thawing stage, the ice lenses dissolve, causing the soil particles to show signs of rehydration. The repeated freeze-thaw cycles result in alterations in the soil porosity and softening of soil particles, causing a gradual decline in both thermal conductivity and volumetric heat capacity, as observed in Fig 6. This behavior can be attributed to the frost actions that occur during the freezing phase, which cause a trend of isolation in soil particles. Meanwhile, the thawing process allows for a partial resumption of these isolated particles.

### 4.3 Prediction and assessment

#### 4.3.1 Baseline model evaluations

The baseline models are categorized into Groups A and B. Note that the proposed model has been assigned B-4 in the second group.

**Group A**: traditional machine learning-based approaches, including:

A-1) XGBoost: is optimized by gbtree mode, with the max depth being two and the eta being 1. The whole operation is running with four parallel threads. Other configurations are the same as those in ref. [61].

A-2) Decision Tree: The configurations are the same as those in ref. [61].

A-3) Random Forest: One hundred estimators are constructed to enhance the performance, while other configurations are the same as those in ref. [61].

A-4) SVR (support vector regression): the radial basis function (RBF) is selected as the kernel function. The error terminating the training process (tol) is set to 0.001. The parameter gamma is set to scale, and the other configurations are the same as those in ref. [61].

**Group B**: deep neural network-based approaches, including:

B-1) the original DNN architecture is constructed based on 3 FC layers with 16, 8, and 2 neurons. This model serves as the baseline of the deep neural network-based approach.

B-2) Based on B-1, the embedding module is integrated into the model, in which only the concatenation operation is applied to fuse the embedding vectors.

B-3) Based on B-1, the attention-based block is applied to fine-tune the learned features.

B-4) Based on B-2, the proposed attention-based block is applied to fuse the embedding vectors. The configuration of B-4 is also the full architecture of the proposed model.

**Fig 7:**
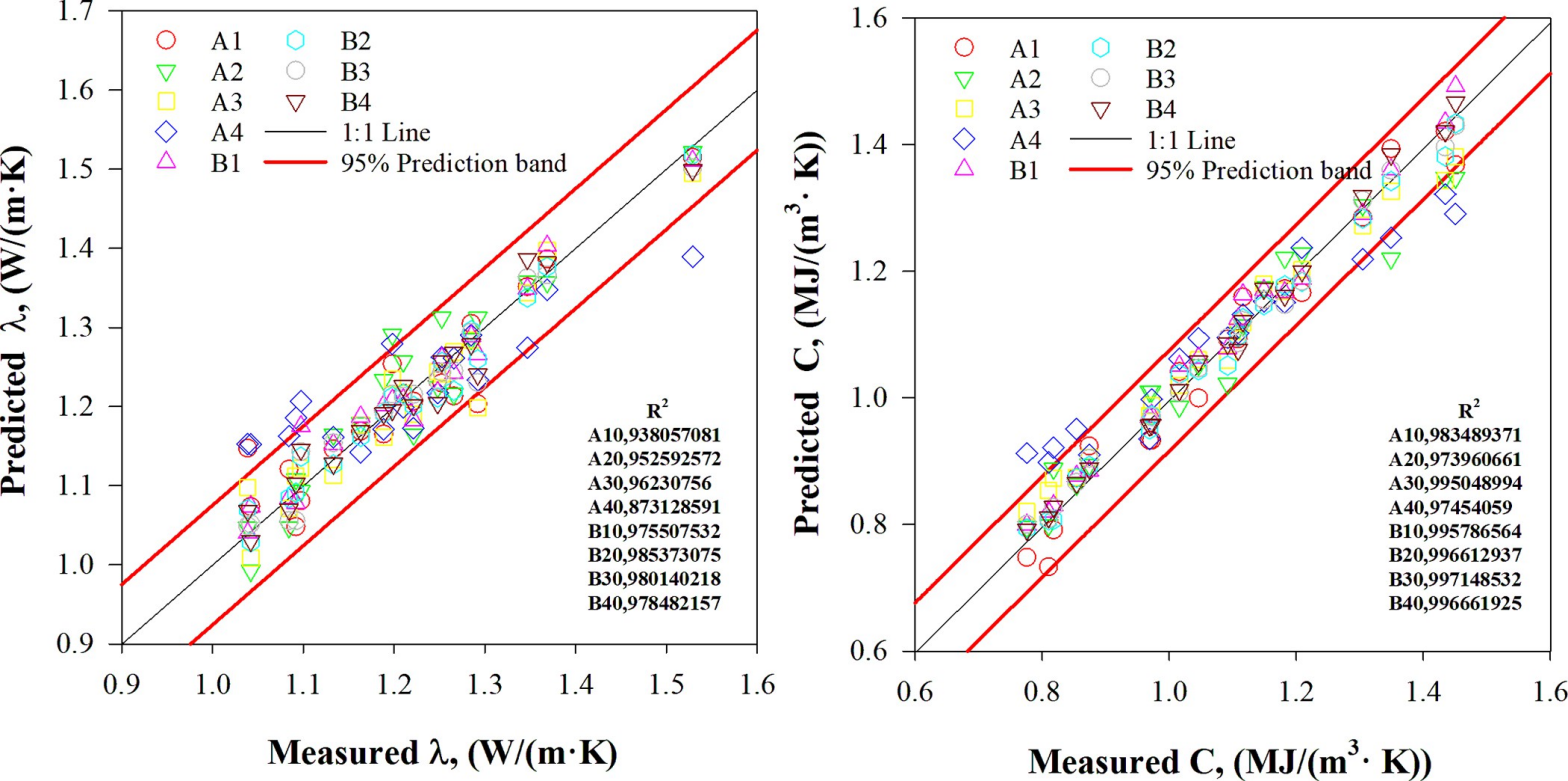
Comparison between predicted and actual data across test sets for baseline models.

The performance of the baseline models on the test sets is depicted in Fig 7. The *EFAttNet*-based predictive model exhibits superior predictive capability compared to the other baseline models, primarily due to the integration of multiple modules in contrast to a traditional deep neural network. Despite the intricate influence of freeze-thaw cycles on soil thermal performance, the evaluation metrics attest to the effectiveness of the *EFAttNet* algorithm in accurately estimating soil thermal properties subjected to such actions.

#### 4.3.2 Assessment of thermal parameter models

**Fig 8:**
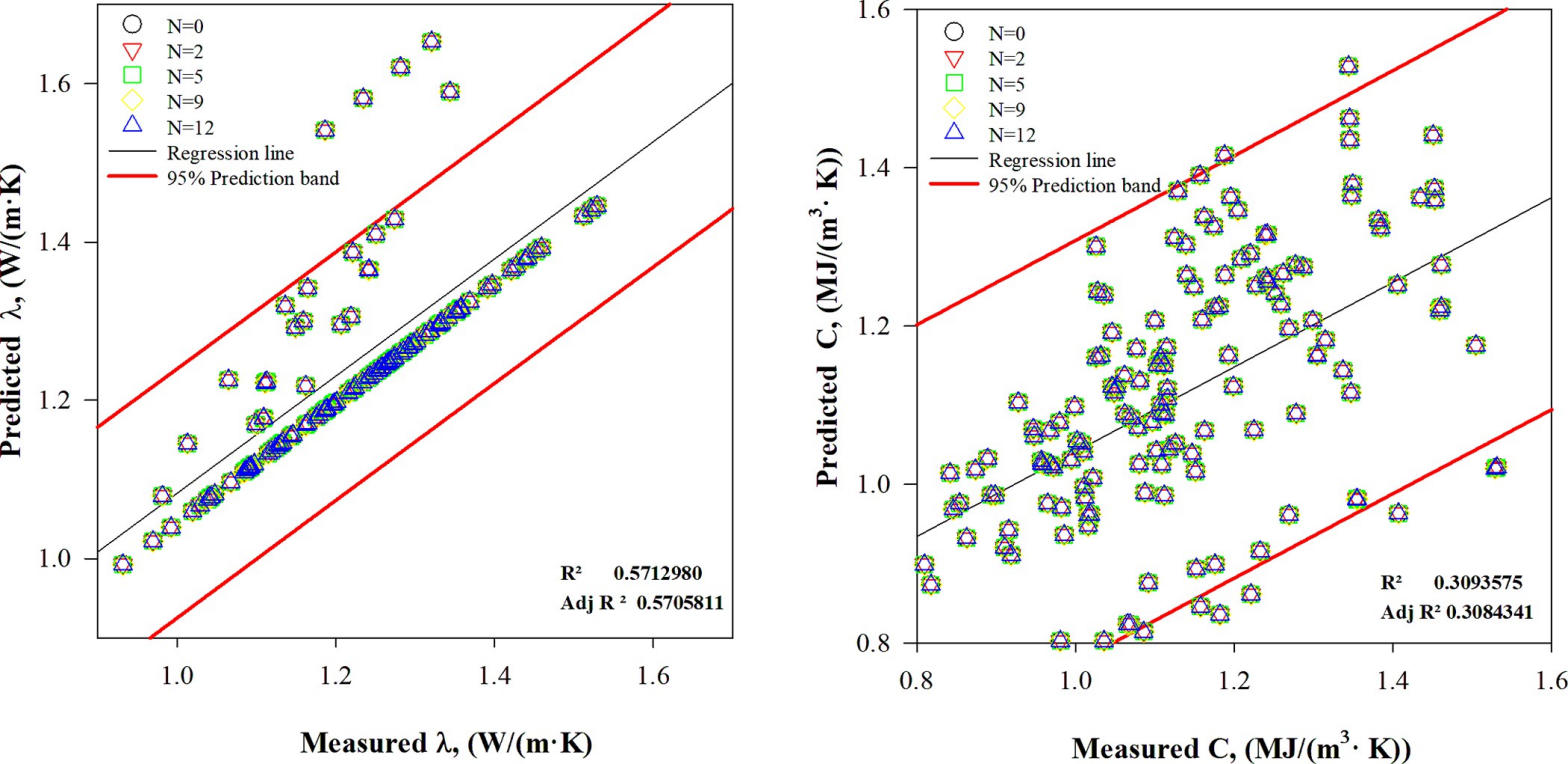
Thermal property evaluation using the *de Vries* model.

Various models’ thermal properties were assessed, and the *de Vries* empirical model’s effectiveness was compared to baseline models. The *de Vries* model outperformed other empirical models in predicting thermal properties under freeze-thaw cycles and low temperatures. Fig 8 compares the measured and predicted thermal properties values using the *de Vries* model is displayed for different numbers of freeze-thaw cycles.

The *de Vries* model exhibited a greater scatter and wider prediction band (95%) in comparison to the deep learning model’s results, as illustrated in Fig 7. This variation could be attributed to the intricate structure of the soil and the limitations in input parameters for the *de Vries* model. This model’s validity is restricted to particular soil moisture content and especially low moisture levels. As soil moisture increased, predictions from the *de Vries* model could become less accurate. It is evident that the *de Vries* model does not reflect experimental outcomes well, owing to distinct temperature gradients and continuous heat sources applied to granular soils. In contrast, deep learning models showed superior ability to predict thermal properties precisely across different conditions.

To evaluate the performance of the proposed model, the other baseline models, and the *de Vries* models for a regression task, three metrics are typically used. These metrics are commonly used to measure the performance of the two predicted items, namely the thermal conductivity and the heat capacity.

1) RMSE (root mean square error)

$$RMSE=\sqrt{\frac{1}{m}\sum_{i=1}^{m}{\left(y_{i}-{ŷ}_{i}\right)}^{2}}$$ (19A)

2) MAE (mean absolute error)

$$MSE=\frac{1}{m}\sum_{i=1}^{m}\left|\left(y_{i}-{ŷ}_{i}\right)\right|$$ (19B)

3) MAPE (mean absolute percentage error)

$$MAPE=100\%\times \frac{1}{m}\sum_{i=1}^{m}\left|\frac{\left(y_{i}-{ŷ}_{i}\right)}{y_{i}}\right|$$ (19C)

To enhance the predictive performance of the models, a random 9:1 split was used to generate training and test sets from the available database. A parallel genetic algorithm was applied to optimize the hyperparameters of the algorithms in Group A, while Group B’s deep neural networks utilized the Adam optimizer. Table 3 presents the obtained results, where *λ* denotes the thermal conductivity, and *C* denotes the heat capacity.

**Table 3:** Evaluation metrics for *EFAttNet*-based predictive model outputs.

Group/Model	ID	Metric	*λ*,	*C*,
			W/(m·K)	MJ/(m^3^·K)
Group A	A-1	RMSE	0.0420	0.0386
		MAE	0.0309	0.0319
		MAPE	2.6351	3.0886
	A-2	RMSE	0.0389	0.0587
		MAE	0.0308	0.0379
		MAPE	2.5889	3.3481
	A-3	RMSE	0.0327	0.0369
		MAE	0.0243	0.0294
		MAPE	2.0470	2.7783
	A-4	RMSE	0.0698	0.0764
		MAE	0.0556	0.0609
		MAPE	4.7105	5.8886
Group B	B-1	RMSE	0.0275	0.0209
		MAE	0.0204	0.0179
		MAPE	1.7125	1.7009
	B-2	RMSE	0.0222	0.0157
		MAE	0.0176	0.0139
		MAPE	1.5118	1.2864
	B-3	RMSE	0.0250	0.0215
		MAE	0.0185	0.0159
		MAPE	1.5426	1.4316
	B-4	RMSE	0.0215	0.0177
		MAE	0.0173	0.0128
		MAPE	1.4354	1.1634
*de Vries* Model	—	RMSE	0.0894	0.1611
		MAE	0.0002	0.0001
		MAPE	4.5491	9.9469

**Note**: RMSE: Root Mean Square Error; MAE: Mean Absolute Error; MAPE: Mean Absolute Percentage Error

*λ*: Thermal Conductivity; *C*: Volumetric Heat Capacity.

Figs 7 and 8 illustrate the accuracy of the developed model and the empirical model proposed by *de Vries*, by showing the *R*^2^ values. It is clear that the thermal properties predicted by the *EFAttNet*-based predictive model are highly consistent with the measured values. The values of the coefficient of determination and the corresponding root mean square error range between 0.9381 and 0.9853, and 0.0215 and 0.0697, respectively, for the thermal conductivity values predicted by the *EFAttNet*-based predictive model. Conversely, for the *de Vries* empirical model, the *R*^2^ and corresponding RMSE values range between 0.5712 and 0.0894. Furthermore, for the heat capacity values, the *R*^2^ and corresponding RMSE values fluctuate between 0.9739 and 0.9972, and 0.01569 and 0.0764, respectively, for the *EFAttNet*-based predictive model. Conversely, for the *de Vries* empirical model, the R^2^ and corresponding RMSE values range between 0.3093 and 0.16108.

The statistical analysis of the developed model demonstrates its superior performance in predicting *λ* and *C* compared to the existing empirical model, as evidenced by the *R*^2^ values being near 1.0 (*R*^2^>0.90 for the *EFAttNet*-based predictive model), and the low RMSE and MAE values.

**Fig 9:**
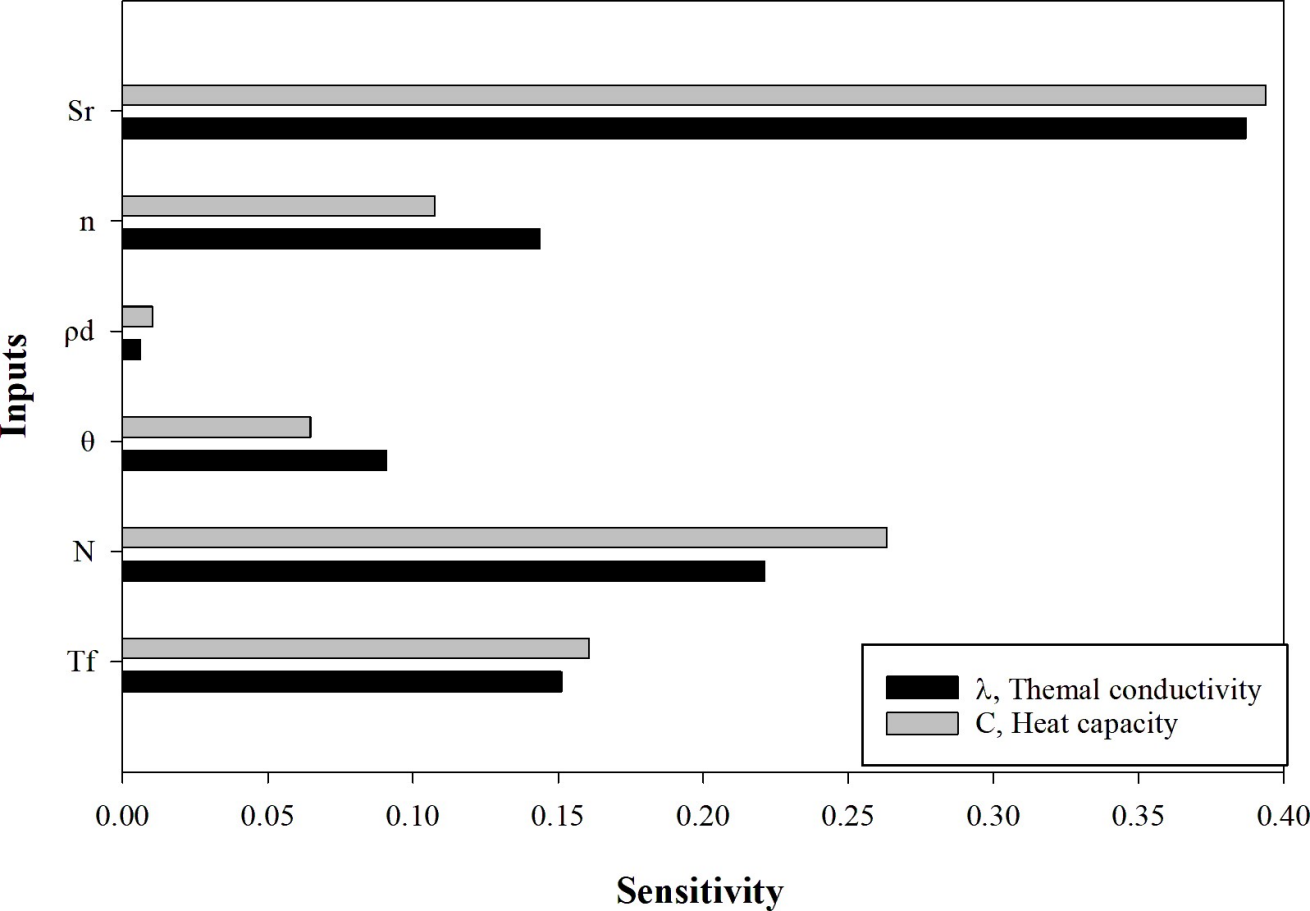
Impact of input variables on outputs: *T*_f_, temperature; *N*, number of F-T cycles; *θ*, moisture content; *ρ*_d_, dry density; *n*, porosity; *S*_r_, degree of saturation.

The goal of a sensitivity analysis is to identify the sources of input uncertainty that may impact the output of a mathematical model [62]. The relative importance of each input on the final predictions of thermal conductivity and heat capacity are shown in Fig 9. The degree of saturation is the most influential factor on the outputs, followed by the number of freeze-thaw cycles, subzero temperature, porosity, moisture content, and dry density. Although the two outputs share a similar pattern regarding the relative importance of each variable, there is a slightly larger difference between predictors for the heat capacity. The effect of freeze-thaw cycles is significant, second only to the degree of saturation, in affecting the evolution of soil thermal properties, which should be considered in the design of earth structures in cold regions. Based on the analysis, it is suggested that the predictor of soil dry density may be removed when conducting a multivariate regression analysis on the acquired data, as its contribution to soil thermal properties is negligible.

## 5. Conclusions

This study conducted experimental assessments of the thermal conductivity (*λ*) and volumetric heat capacity (*C*) of sandy soil specimens under varying conditions, including freeze-thaw cycles at above and subzero temperatures and different moisture levels. To estimate these thermal properties, we developed a predictive model, *EFAttNet*, which utilizes a custom-designed embedding and attention-based fusion network. This model exhibited superior accuracy compared to traditional *de Vries* empirical models and other baseline algorithms.

Initial measurements showed that *λ* values increased linearly with moisture content and decreased with temperature. Conversely, *C* values exhibited a rising trend with both moisture content and freezing temperature. After the freeze-thaw cycles, both *λ* and *C* were positively influenced by moisture content and freezing temperature. In terms of predictive accuracy, the *EFAttNet*-based model excelled, particularly in capturing the nonlinear relationships among various influencing factors. Among these, the degree of saturation had the most significant impact, followed by the number of freeze-thaw cycles, subzero temperatures, porosity, and moisture content. Notably, dry density had a negligible impact on thermal properties, likely due to the overriding effects of other factors or specific characteristics of the studied soils, such as particle size distribution or mineralogical composition.

The findings hold significant implications for construction and engineering projects, particularly in enhancing sustainability and energy efficiency. The accurate prediction of thermal properties aids engineers in determining the appropriate insulation requirements for foundations, thereby impacting the overall energy efficiency of buildings. By integrating the *EFAttNet*-based predictive model into foundation design processes, engineers can optimize insulation strategies and minimize heat transfer, reducing energy consumption for heating and cooling. Additionally, this knowledge is crucial for the design and optimization of geothermal energy systems, as sandy soils, commonly encountered in such projects, require accurate thermal property assessments for efficient operation.

Furthermore, understanding how soil thermal properties vary with moisture content, temperature, and freeze-thaw cycles is vital for mitigating temperature fluctuations’ effects on infrastructure integrity. The insights gained from the *EFAttNet*-based model enable engineers to predict thermal stresses on infrastructure components more accurately and implement measures to enhance durability and resilience. Moreover, these findings may inform the selection of suitable construction materials and landscaping strategies to optimize thermal performance in urban areas. In addition to its implications for construction and engineering, understanding soil thermal properties is essential for conducting accurate environmental impact assessments. Changes in land use or infrastructure projects can alter soil thermal dynamics, potentially affecting local ecosystems and biodiversity. By incorporating detailed knowledge of soil thermal properties into environmental assessments, policymakers and stakeholders can make informed decisions to minimize adverse environmental effects while promoting sustainable development.

The demonstrated predictive accuracy of the *EFAttNet*-based model in estimating thermal properties of soils under various conditions holds promise for practical applications. While the study focused on specific soil types and conditions, the insights gained may guide further research and development in this field, enhancing our understanding and management of soil thermal properties across diverse environments.

## Supporting information

S1 File Inclusivity in global research questionnaire. (DOCX)
